# Physicians’ Moral Dilemmas in the Age of Viagra

**DOI:** 10.1093/geroni/igaa057.3015

**Published:** 2020-12-16

**Authors:** Liat Ayalon

**Affiliations:** Bar-Ilan University, Ramay Gan, HaMerkaz, Israel

## Abstract

Oral phosphodiesterase5 inhibitors (PDE5i; e.g., Viagra®) have become the first line of treatment of erectile dysfunction in men. Relying on interviews with 38 physicians, this study explored moral dilemmas associated with the prescription of PDE5i to older men. Moral dilemmas at the micro level concerned the interest of the patients to receive medical treatment, even when this was counter-indicated. At the meso level, physicians expressed their concerns about the impact of PDE5i on their patients’ partners. At the macro level, physicians discussed the substantial contribution of the pharmaceutical industry to the education of patients and physicians about pharmacological treatments for sexual problems. Physicians reported no moral concerns about industry involvement and reported only the benefits associated with it. The study raises moral issues associated with the treatment of erectile dysfunction. As such, it stresses the importance of facilitating a bio-psycho-social approach to treat sexual dysfunctions.

